# Does the Belief That Contraceptive Use Causes Infertility Actually Affect Use? Findings from a Social Network Study in Kenya

**DOI:** 10.1111/sifp.12157

**Published:** 2021-07-13

**Authors:** Erica Sedlander, Jeffrey B. Bingenheimer, Shaon Lahiri, Mary Thiongo, Peter Gichangi, Wolfgang Munar, Rajiv N. Rimal

**Affiliations:** ^1^ Erica Sedlander, Department of Prevention and Community Health, Milken Institute School of Public Health The George Washington University, Washington, DC, USA. University of California, San Francisco, Department of Family and Community Medicine San Francisco CA USA; ^2^ Jeffrey B. Bingenheimer, The George Washington University, Milken Institute School of Public Health Department of Prevention and Community Health Washington DC USA; ^3^ Shaon Lahiri, The George Washington University, Milken Institute School of Public Health Department of Prevention and Community Health Washington DC USA; ^4^ Mary Thiongo, International Center for Reproductive Health Kenya Mombasa Kenya; ^5^ Peter Gichangi, Technical University of Mombasa, Mombasa, Kenya. Department of Public Health and Primary Care, Faculty of Medicine and Health Sciences Ghent University Ghent Belgium; ^6^ Wolfgang Munar, Department of Global Health, Milken Institute School of Public Health The George Washington University Washington DC USA; ^7^ Rajiv N. Rimal, Department of Health, Behavior and Society Johns Hopkins University Baltimore MD USA

## Abstract

The belief that contraceptive use causes infertility has been documented across sub‐Saharan Africa, but its quantitative association with actual contraceptive use has not been examined. We collected and analyzed sociocentric network data covering 74 percent of the population in two villages in rural Kenya. We asked respondents to nominate people from their village (their network), and then we matched their network (alters) to the individual participant (ego) to understand how their beliefs and behaviors differ. We asked about contraceptive use and level of agreement with a statement about contraceptive use causing infertility. We calculated the average nominated network contraceptive use score and the average nominated network belief score. Holding the individual belief that contraceptive use causes infertility was associated with lower odds of using contraceptive (AOR = 0.82, *p* = < 0.01); however, when one's own nominated network connections held this belief, the odds of using contraceptive were even lower (AOR = 0.75, *p* <0.01). Our findings show that this belief is associated with lower odds of contraceptive use and highlights the role that other people in one's network play in reinforcing it. Sexual and reproductive health programs should address this misperception at the individual and social network level.

## BACKGROUND

Unmet need for family planning—defined as the percentage of women who are not currently using a method of contraception and want to stop or delay childbearing—is high throughout sub‐Saharan Africa (Bradley et al 2012; Sedgh and Hussein 2014). Being fearful about contraceptive use‐related side effects, whether real or perceived, is an important barrier for family planning uptake. A 2014 systematic review that used Demographic Health Survey (DHS) data from over 50 countries found that the most common reason for not using contraception was concern about “side effects or health concerns” (Sedgh and Hussein 2014). These concerns accounted for 28 percent of nonuse in Africa. In Kenya, the site for the current study, these concerns accounted for 43 percent of the variance in nonuse. An important limitation of that study was that the category “side effects and health concerns” included a wide range of factors, including scientifically established side effects as well as myths and misconceptions.

Within this broad category of misperceptions, the belief that contraceptive use causes infertility has been cited in studies across sub‐Saharan Africa (Adongo et al. 2014; Hindin et al. 2014; Sedlander et al. 2018; Ochako et al. 2015; Klinger and Asgary, 2017; Schwandt et al. 2015; Farmer et al. 2015; Gueye, Speizer, Corroon and Okigbo, 2015; Schwartz et al. 2019; Bornstein et al. 2020; Morse et al. 2014; Wasti et al. 2017). A 2020 scoping review of fear of infertility in Africa found 15 qualitative studies that cited that contraception causes infertility. Studies reported that men and women believed that contraception stays in the body and blocks blood and that it can cause structural damage to a woman's reproductive organs. This belief is not only present but also has serious ramifications. A systematic review of barriers to contraceptive use among young people in low‐to‐middle income countries reported that the belief that contraceptive use would cause infertility was the most cited reason for nonuse (Williamson et al. 2012). Otiode, Oronsaye, and Okonofua (2001) found that Nigerian adolescents believed so strongly that modern contraception causes infertility that they preferred abortion to using contraception. Gebremariam and Addissie (2014) found that the belief that contraceptive use causes infertility is significantly associated with intention to use long‐acting reversible contraceptives in Ethiopia. To our knowledge, however, no studies have examined whether the belief that contraceptive use causes infertility is quantitatively associated with actual use. Assessing the existence and strength of that association is the first goal of the current study.

To do this well, we need to account for other factors that are associated with contraceptive use. Past research illustrates that contraceptive use is associated with factors at multiple levels, including the individual level (e.g., age, education, religion, and attitudes), interpersonal level (e.g., perceived descriptive and injunctive social norms around family planning, husband's support for family planning), structural level (e.g., interaction with the frontline health worker system), and socionormative level (Eggers et al. 2016; Worku et al. 2019; Ba et al. 2019; Gebre‐Egziabher et al. 2017). The socioecological model (Bronfenbrenner, 1989) suggests that individual‐level beliefs and attitudes are far from the only determinants of behavior. Individual‐level beliefs about infertility and community‐level social norms that influence contraceptive use may diffuse within villages through one's social network through interpersonal communication (Rutenberg & Watkins 1997; Casterline 2001). Other studies have also found that collective norms around contraceptive use (the prevalence of an attitude, belief, or behavior within a group or community) affect individual use (Sedlander and Rimal 2019). As we examine the association between the belief that modern contraception causes infertility on actual modern contraceptive use, it is important to account for multilevel influences on contraception use.

Since the 1990s, social networks have also been central to our understanding of the spread of contraception in low income and emerging economies. Prior research shows that social networks and social interactions may be critical mediators of the diffusion of contraception. What actually spreads directly from person to person are not the behaviors themselves, but rather beliefs, attitudes, and norms related to fertility and contraceptive use (Casterline 2001; Axinn & Yabiky 2001; Mason 1997). Few studies, however, have had the type of social network data that is necessary to understand these processes. This study is unusual in having data about (a) the identities of respondent's network partners and (b) the self‐reported beliefs and attitudes of those network partners (including the belief that contraceptive use causes infertility). We used a sociocentric approach and attempted to interview the full population of men and women of reproductive age in two villages in Kilifi County, Kenya. Sociocentric network studies attempt to represent the entire network by collecting data on the social ties between all individuals within a defined population (e.g., a village) (Marsden 1990). This type of data increases our understanding of the mechanisms by which social networks may affect attitudes and behaviors (e.g., contraceptive use) (Perkins, Subramanian, and Christakis 2015). A socionetwork approach enabled us to examine if one's nominated social networks’ modern contraceptive use is associated with their own individual use. These data also allowed us to examine if one's nominated social networks’ beliefs that modern contraceptive use causes infertility was associated with their own contraceptive use. With more calls to move beyond examining individual factors, this approach sheds light on multilevel contraceptive influences (Shulman et al. 2017; Sedlander and Rimal 2019). The second goal of the current study, therefore, is to use those data to test the hypothesis that the prevalence and strength of the belief that modern contraceptive use causes infertility among one's social network partners is associated with one's own use.

Our prior qualitative research in this part of Kenya showed that the belief that contraceptive use causes infertility was particularly acute for younger women before they proved their fertility by having children (Sedlander et al. 2018). In other words, they felt pressure to “assess their fertility” before using contraception so once this is proved (after giving birth), they may be more likely to use contraception despite lingering fears that it may cause secondary infertility. Primary infertility is defined as the inability to conceive after 12 months of regular unprotected sex, and secondary infertility refers to this among women who have already given birth (WHO, 2020). Our prior qualitative data also showed that participants believed that contraception can particularly “harm” younger and unmarried women and “make your womb weak” (Sedlander et al. 2018). The third goal of the current study, therefore, is to test the hypotheses that the association between the belief that modern contraceptive use causes infertility and actual use of modern contraception will be (a) stronger among younger women than among older women and (b) stronger among nulliparous women than among women who have had at least one child.

## HYPOTHESES

*H1*: The belief that contraceptive use causes infertility (at the individual level) will be negatively associated with modern contraceptive use.
*H2*: The belief that modern contraceptive use causes infertility among one's network contacts (network beliefs) will be negatively associated with respondent's modern contraceptive use.
*H3*: Being older (compared to younger) will attenuate the relationship between the belief that modern contraception causes infertility and contraceptive use (both at the individual and network belief level).
*H4*: Having children (versus not) will attenuate the relationship between the belief that modern contraception causes infertility and contraceptive use (both at the individual and network belief level).

## METHODS

### Selection of Study Communities

The setting for the overall study from which we collected this data is Kilifi County, Kenya, which lies directly north of Mombasa along the Indian Ocean. Kilifi has some of the highest poverty levels, lowest literacy rates, and highest indicators of gender inequity nationally (Molyneux, Peshu, and Marsh 2005; Molyneux et al. 2007). The total fertility rate is 5.1 children per woman, 99.7 percent of women have heard of contraception, and 34.1 percent of currently married women ages 15–49 use contraception (Kenya National Bureau of Statistics 2014). Compared to Kenya as a whole, Kilifi County has higher fertility and lower use of contraception. Overall, the total fertility rate in Kenya is 3.9 children per woman and 58 percent of married women are using contraception (Kenya National Bureau of Statistics 2014).

Our study focused on two rural communities within Kilifi County, which we selected on the basis of modern contraceptive prevalence as estimated in recent rounds of the Performance, Monitoring, and Evaluation 2020 (PMA 2020) survey program. In one, the modern contraceptive prevalence among women of childbearing age had recently been estimated at 44.4 percent—a relatively advanced stage of fertility transition. The other was intended to represent an earlier stage of that transition, and its contraceptive prevalence was estimated at 10.3 percent. Both are inland rather than on the coast, but the higher contraceptive prevalence community is closer to and more accessible from the coast road than the lower modern contraceptive prevalence community.

### Data Collection Procedures

In spring 2018, data were collected using *Trellis* (https://trellis.yale.edu/), a mobile application developed for the collection of social network data in hard‐to‐reach locations (see Lungeanu et al. 2021 for a more detailed description of Trellis). After a two week in person training, research assistants from the International Center for Reproductive Health‐Kenya (ICRH‐K) first compiled a roster of all residents aged 15 or older in the two study villages. The higher contraceptive use village was much larger in size (*n* = 1,735 eligible participants) compared to the lower contraceptive use village (*n* = 875 eligible participants). Next, given that many people in the villages had the same names, once informed consent was obtained, photos of residents were taken using Android phones and were then uploaded into Trellis along with the roster data (69 percent of all residents from the roster had their photo taken). Trellis issued unique identifiers for each respondent that were matched to their photos and demographic information. Their respective photos were used during the subsequent step, survey data collection, to identify their social network contacts (alters) and to confirm their identity. Trellis pulled this information from the predefined list (the roster dataset) and in cases that the person they named or described was missing in the dataset, research assistants added the new names/contacts at that point (*n* = 123).

The overall response rate was 72 percent of the total villages (71.5 percent from the higher contraceptive use village and 72 percent from the lower contraceptive use village), with 66 percent of the nonresponse being due to the study team being unable to locate the person, and the remaining 34 percent being due to refusal. Participants did not receive compensation for their participation in the study. The project was reviewed and approved by the Institutional Review Boards at the three participating universities: George Washington University, Northwestern University, and Kenyatta National Hospital/University of Nairobi's Ethics and Research Committee.

### Eligibility Criteria

Men or women between the ages of 15 to 49 who are currently married or living with a partner were included in this study. We chose to only include participants ages 15–49 because they are considered to be of reproductive age. While data collection included men and women who were not married and cohabitating, for this analysis, we chose to include only married and cohabitating participants because they are more likely to be sexually active. While participants who were not married or cohabitating were excluded in primary analysis, they were analyzed separately in a sensitivity analysis. After only including participants who met our inclusion criteria, our total sample included 918 participants, of which 273 were from the lower contraceptive‐use village and 645 were from the higher contraceptive‐use village. As previously noted, the difference in sample size from each village reflects the size of each village, not the response rate.

### Measures

For the demographic variables, we measured parity as a binary variable (has at least one child or not), age was measured as continuous numerical measure, education was a categorical question asking about the highest level of education completed, religion was measured as a binary variable (Muslim or other), and marital status was binary (married/cohabitating or not).

We measured the belief that contraception causes infertility as the level of agreement (on a five‐point scale) to the statement “Using modern methods of family planning can cause a woman to become infertile” (higher scores represented greater agreement).

Following Geber et al. (2019), we also conceptualized network beliefs in terms of beliefs and behaviors among those in one's close social network. In the questionnaire, we asked participants to nominate up to five people with whom they spend a lot of time. Participants could nominate anyone but we only included nominees in the analysis who fit our eligibility criteria (e.g., ages 15–49). There are many ways to elicit social network membership, and each may result in different network members. For example, the people who you borrow money from may be different from the people you spend a lot of time with. We chose the question, “with whom do you spend a lot of time,” after discussions with our in‐country data collection partners and pilot testing. Participants mentioned up to five people and reported their relationship to them (e.g., friend, sister, husband, etc.). Some individuals nominated more reference group members than others (range 1–5). We restricted our sample to those who nominated at least one reference group member. Thus, some people mentioned only one person that they spent a lot of time with. For example, a husband might only list his wife. If people mentioned fewer network members (e.g., two vs. four) then those two network members would have more weight in the network contraceptive use measure than four members. Some participants nominated network contacts who did not participate in the survey, either because (a) they were not residents of the village or (b) they were residents but either could not be located for an interview or declined to be interviewed, or (c) they did not fit our eligibility criteria. To calculate network beliefs, the average belief that contraception use causes infertility, we took the response for each of the nominated members and averaged them to create a unique network belief measure for each person (range = 0–1). A higher mean from one's social network signified that more people within their network agreed with this belief. For example, a mean of 4 shows that on average, one's nominated network “agrees” with the statement that “Using modern methods of family planning can cause a woman to become infertile.”

Based on cognitive interviews in the community, we decided to use the term “medical methods of family planning” in place of modern contraception. We measured modern contraceptive use with one question, “Are you or your partner currently using medical methods of family planning to delay or avoid having a child? Remember by medical methods of family planning we mean injectables, implants, the pill, condoms, the IUD, and sterilization.” We specifically asked about “your or your partner” to include both men and women in the analysis. We then created a binary variable for modern contraceptive use. We then ran a sensitivity analysis that only included participants who subsequently reported that they were using hormonal methods (injectables, implants, the pill, and emergency contraception). As previously mentioned, prior research shows that the belief that contraception causes infertility is primarily focused on hormonal methods.

We measured network contraceptive use with the same approach that we used to measure network beliefs. We took the modern contraception use score for each of the nominated members and averaged them to create a unique network contraceptive use measure for each person (range = 0–1). For example, if someone nominated two people and one used modern contraception and the other did not, that person's network contraceptive use score would be 0.50 (half the people in their nominated social network used contraception). If they nominated five people and all of them use contraception, then their score would be 1 (all people in their nominated social network use contraception).

We measured awareness about contraceptive methods with the following question, “have you ever heard of the following family planning methods?” (Check all that apply). Modern method options included: contraceptive implant, IUD & “the coil,” injectables, the pill, female sterilization, and male sterilization. Response options were “yes” or “no.” We created a composite score to reflect the methods that women were familiar with. A higher score indicates greater awareness about different modern contraceptive methods.

To account for factors at the interpersonal level that prior research shows are associated with modern contraceptive use, we measured both descriptive and injunctive norms around modern contraceptive use. Descriptive norms are people's perceptions about the prevalence of the focal behavior. We measured perceived descriptive norms around contraceptive use with two questions (*r* = 0.84): “Most people around me use medical methods of family planning for determining when to have a child” and “Most people whose opinions I value use medical methods of family planning for determining if or when to have a child.” Response options were on a five‐point Likert scale and ranged from strongly disagree to strongly agree, higher responses being coded as greater agreement.

Injunctive norms refer to people's perceptions about social sanctions they will experience if they do not conform to others’ beliefs. Similar to descriptive norms, we measured perceived injunctive norms with two questions (*r* = 0.75): “Most people important to me will think badly of me if I use medical methods of family planning and “most people important to me will reject me if I use medical methods of family planning.” Response options were also on a five‐point Likert scale and ranged from strongly disagree to strongly agree, higher responses being coded as greater agreement that others *disapprove* of family planning. We also asked about age, marital status, number of children, religion, and education.

## ANALYSIS

We conducted our analyses in three steps. First, we calculated descriptive statistics including *t*‐tests to compare the belief that contraceptive use causes infertility among the two villages, higher and lower modern contraceptive prevalence. We then performed bivariate Pearson's zero‐order correlations to describe how this belief is associated with other multilevel factors. Finally, we used nested multivariable logistic regression analyses to examine whether the belief that contraceptive use causes infertility is associated with actual contraceptive use and to test for interactions. We used Stata 14 to conduct all analyses.

For the individual‐level logistic regression, we included the following variables in the model: age, marital status, religion, education, whether or not participants had children, awareness of different family planning methods, and belief that using contraception makes a woman infertile. For the interpersonal‐level model, we added perceived descriptive and injunctive social norms around family planning. For the community‐level model, we included network contraceptive use and network belief that contraception use causes infertility. Finally, we included all previously mentioned variables and examined four interaction terms in four separate models: (1) the individual belief that contraception causes infertility moderated by having children, (2) the same individual belief moderated by age, (3) the network belief that contraception causes infertility moderated by having children, and (4) the same network belief moderated by age.

We also conducted two sensitivity analyses. First, we included men and women who were not married or cohabitating. We wanted to ensure that we were not missing an important part of the population because participants who are not married or cohabitating may also be sexually active but we did not have a measure of sexual activity. Second, we included only hormonal methods in how we measured the dependent variable, (modern contraceptive use). In Boivin et al. (2020)’s systematic review of fear infertility in Africa, authors found that the majority of studies reported that hormonal methods (e.g., injectables and oral contraceptives) would cause infertility. Only one study cited the perception that condom use could cause infertility. For the sensitivity analysis, we limited hormonal methods of contraception to the implant, injectables, the pill, and emergency contraception. We chose not to include IUDs (*n* = 16), because the majority of IUDs in this region, are nonhormonal (Marie Stopes International ‐ Kenya, n.d.).

## RESULTS

Descriptive statistics for the sample are presented in Table 1. The average age of men and women in both villages was approximately 32 years. About 38 percent of the sample from both villages were Protestant/Catholic and 53 percent were Muslim. Marital status was approximately the same in both villages with about 84 percent of men and women in monogamous marriages, 10 percent in polygamous marriages, and 5 percent living together but not married. About 94 percent of the sample in both villages had at least one child (range 0–15, mean 3.85). Overall, the two study villages were more similar in their contraceptive use than we anticipated. However, contraceptive use was lower in the lower modern contraceptive prevalence village at 31 percent compared to 52 percent in the higher modern contraceptive prevalence village. The belief that modern contraceptive use causes infertility was more prevalent in the lower modern contraceptive prevalence village with 66 percent of respondents believing it, compared to 60 percent in the higher modern contraceptive prevalence village. We ran independent sample *t*‐tests to compare this belief by village. There was a small but significant difference in the scores for the belief in the lower contraceptive prevalence village (M = 3.66, SD = 0.072) compared to the higher contraceptive prevalence village (M = 3.42, SD = 0.04); *t* (−3.06) (*p* = 0.002).

**TABLE 1:** Description of the Sample (married/living together men and women ages 15‐ 49 years old)

	Combined village	Higher contraception village	Lower contraception
	*M (SD)*		
Age	32.1 (8.54)	32.08 (8.49)	32.21 (8.62)
Education	*n* (%)	*n* (%)	*n* (%)
None	214 (23.31)	122 (18.91)	92 (33.70)
Primary	490 (53.38)	373 (57.83)	117 (42.86)
Secondary	98 (10.68)	78 (12.09)	20 (7.33)
Religion			
Roman Catholic	15 (1.63)	6 (0.93)	9 (3.30)
Protestant/other Christian	347 (37.80)	207 (32.09)	140 (51.28)
Muslim	489 (53.27)	428 (66.36)	61 (22.34)
Has children			
Yes	867 (94.44)	609 (94.42)	258 (94.51)
No	51 (5.56)	36 (5.58)	15 (5.49)
Marital status			
Married (monogamous)	773 (84.20)	543 (84.19)	230 (84.25)
Married (polygamous)	91 (9.91)	64 (9.92)	27 (9.89)
Living with a man/woman	54 (5.88)	38 (5.89)	16 (5.86)
Currently using contraception	423 (46.08)	337 (52.25)	86 (31.50)
Believes family planning causes a woman to become infertile	572 (62.44)	391 (60.81)	181 (66.3)

NOTES: Believes family planning causes a woman to become infertile is dichotomous. Agrees or strongly agrees = believe.

Table 2  shows the zero‐order correlations among the variables used in this study combined for both lower and higher contraceptive prevalence villages. In both villages, the individual belief that contraceptive use causes infertility was negatively associated with contraception use (*r =* −0.15). Injunctive norms (social sanctions) not to use contraception (*r =* −0.16,) and network beliefs that contraceptive use causes infertility were also negatively associated with contraceptive use (*r =* −0.18). Network contraceptive use (*r =* 0.20) and descriptive norms that people in one's network were using contraception (*r =* 0.13) were positively associated with individual contraceptive use.

**TABLE 2:** Zero‐order pearson correlations (bivariate associations) of study variables

	Contraception use	Infertility belief	Age	Number of children	Education	Awareness	Descriptive norms	Injunctive norms	Network Contraceptive use	Network beliefs
Contraception use	1.00									
Infertility belief	−0.15***	1.00								
Age	−0.06	0.12***	1.00							
Number of children	0.05	0.01	0.36***	1.00						
Education	0.07*	−0.08*	0.04	−0.13***	1.00					
Awareness	0.14***	−0.00	0.03	0.03	0.12***	1.00				
Descriptive norms	0.13***	0.04	−0.04	−0.00	0.03	0.14***	1.00			
Injunctive norms	−0.16***	0.08^*^	−0.00	−0.03	−0.02	0.00	−0.06	1.00		
Network contraceptive use	0.20***	−0.16***	−0.03	0.05	0.09*	0.04	.10**	−0.02	1.00	
Network beliefs	−0.18***	0.16***	0.04	0.02	−0.09**	−0.05	−0.03	0.10**	−0.22***	1.00

* *p* < 0.05, ^**^ *p* < 0.01, ^***^ *p* < 0.001.

NOTES: Contraceptive use refers to individual contraceptive use. Infertility belief = individual belief that using contraception causes infertility. Awareness = awareness of different modern contraceptive methods. Descriptive norms = perceptions that people around you are using contraceptive. Injunctive norms = expectations that women should *not* be using contraception. Network contraceptive use = the average of modern contraceptive use of the 1–5 people that the respondent nominated. Network beliefs = the average belief that contraceptive use causes infertility of the 1–5 people that the respondent nominated.

The belief that contraceptive use causes infertility was associated with older age (*r* = 0.12, *p* < 0.001), injunctive norms that disapprove of contraception (*r* = 0.08, *p* < 0.05), and network beliefs that contraceptive use causes infertility (*r* = 0.16, *p* < 0.001). Education (*r* = −0.08, *p* < 0.05) and network contraceptive use (*r =* −0.16, *p* < 0.001) were negatively associated with the belief that contraceptive use causes infertility.

For our first hypothesis, we hypothesized that, after controlling for factors at multiple levels, the belief that contraceptive use causes infertility would be negatively associated with individual contraceptive use. Regressions in Table 3 show that this hypothesis was confirmed in two out of three models (individual and interpersonal). People who believe that contraceptive use causes infertility had reduced odds of using contraception themselves in the individual model (AOR = 0.82, *p* <0.01, CI 0.72, 0.93) and in the interpersonal model (AOR = 0.82, *p* < 0.01, CI 0.72, 0.94), but the effect was attenuated when we added in the network‐level variables (AOR = 0.88. *p* > 0.05, CI 0.76, 1.02).

**TABLE 3:** Odds ratios from logistic regression models predicting contraceptive use in married Kenyan men and women (*N* = 823)

	Individual‐level model	Interpersonal‐level model	Network‐level model
Infertility belief	0.82** (CI: 0.71 ‐ 0.93)	0.82** (CI: 0.71 ‐ 0.93)	0.88 (CI: 0.76‐1.01)
Village	0.35*** (0.25 ‐ 0.49)	0.37*** (CI: 0.25 ‐0.51)	0.41*** (CI: 0.28 ‐ 0.58)
Marital status	0.93 (CI: 0.71 ‐ 1.20)	0.92 (CI: 0 .70 ‐ 1.19)	0.92 (CI: 0.70 ‐ 1.21)
Age	0.96*** (CI: 0.94 ‐ 0. .97)	0.96*** (CI: 0.94 ‐ .98)	0.96*** (CI: 0.94 ‐ 0.98)
Number of children	2.12*** (CI: 1.39 ‐ 3.21)	2.10** (CI: 1.35 ‐ 3.16)	1.92** (CI: 1.24 ‐ 2.96)
Muslim religion	0.76 (CI: 0.55 ‐ 1.04)	0.78 (CI: 0.56 ‐ 1.07)	0.81 (CI: 0.58 ‐ 1.13)
Education	1.18** (CI: 1.05 ‐ 1.32)	1.18** (CI: 1.04 ‐ 1.32)	1.14* (CI: 1.01 ‐ 1.29)
Awareness	3.86*** (CI: 1.8 ‐ 8.18)	3.64** (CI: 1.67 ‐ 7.90)	3.19** (CI: 1.42 ‐ 7.10)
Descriptive norms		1.32** (CI: 1.10 ‐ 1.57)	1.33** (CI: 1.10 ‐ 1.59)
Injunctive norms		0.75** (CI: 0.63 ‐ 0.87)	0.74** (CI: 0.61 ‐ 0.87)
Network contraceptive use			2.04** (CI: 1.34 ‐ 3.08)
Network infertility beliefs			0.75** (CI: 0.61‐ 0.91)
Log likelihood	−548.5	−536.3	−500.6
(Pseudo *R*‐squared)	(0.08)	(0.10)	(0.12)

* *p* < 0.05, ^**^ *p* < 0.01, ^***^ *p* < 0.001.

NOTES: Infertility belief = individual belief that using contraception causes infertility. Awareness = awareness of different modern contraceptive methods. Descriptive norms = perceptions that people around you are using contraception. Injunctive norms = expectations that women should *not* be using contraception. Network contraceptive use = the average of modern contraceptive use of the 1–5 people that the respondent nominated. Network infertility beliefs = the average belief that contraception use causes infertility of the 1–5 people that the respondent nominated.

In our second hypothesis, we predicted that network beliefs that contraceptive use causes infertility would be negatively associated with contraceptive use. We found that when one's nominated social network believed that contraceptive use causes infertility, then participants had even more reduced odds of using contraception themselves (AOR = 0.75, *p* < 0.01, CI 0.61, 0.91).

Network contraceptive use and individual contraceptive use were significantly correlated (*r* = 0.20, *p* < 0.001) but also distinct. Similarly, network beliefs and individual beliefs about infertility were significantly correlated (*r* = 0.16, *p* < 0.001) but also distinct.

Our third and fourth hypotheses predicted four different interactions, but none of them were significant. Based on our qualitative research in the same villages, we predicted (H3) that older age would attenuate the association between the individual belief that contraception causes infertility and contraceptive use. We also predicted (H4) that having one or more children would attenuate the association between the individual belief that contraception causes infertility and contraceptive use. Tests show that neither age nor parity interactions were significant. (AOR = 0.99, *p* > 0.05, CI 0.98, 1.01), (AOR = 0.79, *p* > 0.05, CI 0.33, 1.9). Similarly, we also found that age and having children did not attenuate the effect of one's networks beliefs about contraception and individual contraceptive use, (AOR= 1.01, *p* > 0.05, CI 9.99, 1.04), (AOR= 1.63, *p* > 0.05, CI 0.67, 3.99). Given that none of the interactions were significant, they are not included in the tables.

### Sensitivity Analysis

The results of our two sensitivity analyses are shown in Online Appendix Tables 1 and 2. The results presented in Online Appendix Table 1, which include participants regardless of marital status or cohabitation, are largely similar to results from the main analyses. We also find similar results when we code modern contraception (our main outcome) as use of hormonal methods only (Online Appendix Table 2).

## DISCUSSION

In this study, we found that the prevalence of the belief that contraceptive use causes infertility among one's nominated network partners was positively associated with respondents’ own adherence to the belief. Our multivariate models show that this misperception is indeed associated with reduced odds of contraceptive use and that if one's nominated social network holds this belief, it is associated with even more reduced odds of contraceptive use. None of the four interactions tests around age and having children were significant. To our knowledge, this is the first study to examine if this belief is quantitatively associated with actual contraceptive use despite calls to design interventions to address this misperception (Sedlander et al. 2018; Diamond‐Smith 2012; Boivin et al. 2020; Marston and Francis 2019; Harper et al. 2017).

Our findings align with those of others. A study in Senegal, Kenya, and Nigeria (Gueye et al. 2015) examined how misperceptions affected contraceptive use. They created an index of seven to eight misperceptions about modern contraception, including the belief that “use of a contraceptive injection can make a woman permanently infertile” and “contraceptives can harm your womb.” They found that each additional myth that a woman believed (seven to eight total) was associated with lower odds of using contraception. Specifically, in Kenya, a one‐point increase in the number of myths that a woman believed was associated with a 35 percent decrease in her likelihood of using contraception. While they did not isolate the belief of interest in their index, their finding provided evidence of an association between belief and use. Our findings were also in line with another study (Gebremariam and Addissie 2014) that reported that participants who believed that long‐acting and permanent contraceptive methods could “harm a woman's womb” had 76 percent lower odds of *intention* to use them, compared to those who did not hold such belief. Our research takes this one step further and assesses actual use while also controlling for network effects associated with individual use.

We found that network beliefs were significantly associated with behavior even after controlling for perceived descriptive and injunctive norms about contraceptive use. This in line with Geber et al. (2019) who also found that nominated network behaviors influenced risky driving behavior, after controlling for individual attitudes and perceived social norms. As interventionists ponder where to intervene to ensure that men and women have accurate information about family planning, they should note that peer influences might have a larger effect on behavior than individual‐level attitudes and beliefs. Prior qualitative research has indeed shown that beliefs and misperceptions spread from person to person throughout community connections and relations (Sedlander et al. 2018; Rutenberg and Watkins 1997). We propose that these findings call for a multilevel, norms‐based approach to address this misperception, one that includes one's social network as a key target. While contraceptive use and infertility may be considered private behaviors, beliefs held by one's community referents can impact individual behaviors.

Additionally, our bivariate findings and multivariate findings provide evidence about which type of people may be more likely to hold this belief. Specifically, older age and less education are associated with this belief. Interventions may want to consider which age groups and education levels may be more amenable to changing this misperception.

Recently, the Lancet–Guttmacher Commission on Sexual and Reproductive Health and Rights highlighted the need to focus on infertility as a core piece of the sexual and reproductive health agenda (Starrs et al. 2018). While the sexual and reproductive health community has historically focused on family planning, our findings highlight how beliefs about infertility and family planning use are intricately connected. Therefore, a more person‐centered rights‐based approach which includes the whole reproductive life course spectrum from preventing unintended pregnancies to reaching fertility goals is critical. Debunking myths and misperceptions and providing accurate information and resources about the true causes of infertility cannot be ignored. Research needs to address this belief while also examining the mechanisms that trigger it and the social context that perpetuates it (Edberg and Krieger 2020). Prior research shows that inequitable gender norms often hold women more responsible than men for infertility while in reality, the problem is often male related infertility (Irvine 1998). And in rural parts of sub‐Saharan Africa, the social and economic consequences of infertility for women are devastating (Bornstein et al. 2020; Rouchou 2013). Therefore, it should not be surprising that the belief that using contraception may make it impossible to become a mother trumps the risk of an unintended pregnancy. Furthermore, reproductive services are far from equally accessible. Between 49 million and 180 million couples worldwide might be affected by infertility, and services are mainly available only to the wealthy (Starrs et al. 2018). Calls for infertility information and advanced reproductive technology services for all women have been ongoing but have received little traction (Starrs et al. 2018).

To increase contraceptive use and reduce unmet need in rural Kenya and perhaps similar areas of sub‐Saharan Africa, interventionists need to debunk this misperception and given that fertility is so highly valued, educate women on the real causes of infertility (Inhorn 1994; Richards 2002). However, people often do not make rational decisions based on information alone (Elkind 2008). As shown in our models and others, social norms around contraceptive use are powerful (Adams, Salazar, and Lundgren 2013; Sedlander and Rimal 2019). Ample evidence exists indicating that information provision alone (in an effort to improve knowledge) is insufficient for behavior change. To do so will require social norms change as well, which may act as a force multiplier for change efforts. Perhaps social modeling could provide an avenue to change social norms while showing women who have used contraception and still became pregnant. Though not covered in this paper, leveraging the role of key opinion leaders may also be a natural first step for social influence intervention efforts. Community health workers have also been successful in conveying health messages and changing social norms and may be a logical avenue for dispelling myths and misperceptions (Weidert et al. 2017).

## LIMITATIONS

Our study has several limitations. Primarily, our study is limited to two rural communities in Kilifi County, Kenya so it may not be representative of Kenya, or even urban areas of Kilifi County. Another limitation is that our measure of the belief that family planning use affects fertility does not include time to pregnancy. There may be differences in women believing that contraception delays conception versus making it impossible altogether. Future research should examine these nuances as these are two separate beliefs: family planning delays fertility versus causes one to be infertile indefinitely. Additionally, our measure does not include beliefs about different methods of family planning. Beliefs may differ between methods (e.g., the IUD vs. injectables). Future research should compare how beliefs differ by method and timeframe. It is especially important that we measure perceptions about delay in conception after certain contraceptive methods given that long‐term conception is not affected by contraceptive use (Girum and Wasie 2018), but studies are mixed on the effects of some contraceptive methods and short‐term effects on conception (Barnhrt and Schriber 2009). Additionally, we did not ask women why they were not using contraception. They may not be sexually active, they may want to get pregnant, they may be pregnant, they may menopausal, or they may believe they are infertile. Including a question like this would have allowed us to narrow our sample to women who were sexually active, were at risk of getting pregnant, and did not want to get pregnant.

Furthermore, while we interviewed 74 percent of the eligible population, we are missing almost a quarter of eligible participants. There may be systematic differences between those included in the study and those that were not which could bias the results. Additionally, we defined our social network by “quantity of time that you spend with people.” This measure is more generic than we would prefer, as it assumes that one's frequent company comprises the same individuals as one's source of contraception information. It is possible, for example, that one spends a great deal of time with one's mother‐in‐law and yet, for family planning information, one may rely on information from a healthcare provider or distant friend, with whom one spends less time. Nevertheless, our findings show that this measure was significant. Defining a social network based on the behavior of interest may result in an even stronger association. Furthermore, the cross‐sectional design of this study does not provide a strong basis for causal inference. However, the association between modern contraceptive use and the belief that contraceptive use causes infertility persists after controlling for several individual, interpersonal, and social network variables, providing stronger evidence that the association may be causal. However, a longitudinal study could exam whether changes in the belief that modern contraceptive use causes infertility are associated with changes in modern contraceptive use.

Finally, social network analysis is a powerful methodology and this paper did not use all of the available tools. For example, we did not explore centrality measures to find the most influential or connected individuals. We also did not explore concordance and discordance within people's network. We chose to focus on specific research questions to test our hypotheses, but future research using a robust sociocentric dataset could use these tools to inform sexual and reproductive health programs.

Despite these limitations, this study has several strengths. Our hypotheses are based on qualitative findings from the same villages so were able to not only confirm but to build on our previous findings (Sedlander et al., 2018). We used a sociocentric design that enabled us to survey most people—74 percent of residents. We also included both men and women as we know from prior research that men's opinions matter a great deal in a woman's ability to use contraception (Peer et al. 2013; Onwuzurike and Uzochukwu 2001). Finally, the use of social network data enabled us to examine how one's nominated social network beliefs and behavior is associated with their individual modern contraceptive use.

## CONCLUSION

Our findings shed light on the salience of the belief that contraceptive use causes infertility and its association with actual use. We show that not only is one's individual belief associated with individual use but one's social networks beliefs has even more of an association with individual use. From a measurement perspective, this provides additional information about important areas to assess. In late 2019, largely based on the previously cited mounting evidence that the belief that contraception impacts fertility, the United States Agency for International Development (USAID), which administers the DHS, added the response option, “Method could cause infertility” as a more specific choice beyond the existing generic option “method related health concerns” for contraception nonuse. This new measure provides additional information about the percentage of women who choose not to use contraception because of this belief alone. As evidence builds that demand side factors are significant barriers to contraceptive use (Sedgh and Hussein 2014), global health researchers are grappling with how to address them. Our study illustrates the importance of focusing on the belief that contraceptive use causes infertility and points to the fact that multilevel interventions with longitudinal evaluations are likely needed. This evidence should bolster previous calls to include debunking this misperception as part of a comprehensive community‐level strategy to increase family planning use and to reduce unmet need.

## Supporting information

Supporting information

## Data Availability

The data that support the findings of this study are available from the corresponding author upon reasonable request.
