# Supplementary material for: Does the Belief That Contraceptive Use Causes Infertility Actually Affect Use? Findings from a Social Network Study in Kenya
Source: Stud Fam Plann. 2021 Jul 13;52(3):343–59. doi: 10.1111/sifp.12157 (PMC8457152; doi:10.1111/sifp.12157)
Supplement: Supplementary file 1 — Supporting information [file SIFP-52-343-s001.docx]

**Appendix 1 – Sensitivity Analysis – only including hormonal methods as using contraception**

| ***Table 1. Odds ratios from logistic regression models predicting contraception use in married Kenyan men and women (N = 870)*** | | | |
| --- | --- | --- | --- |
|  | Individual-level model | Interpersonal-level model | Network-level model |
| Infertility belief | 0.86* (CI: 0.76-0.97) | 0.86* (CI: 0.76 - 0.98) | 0.94 (CI: 0.81-1.08) |
| Village | 0.34*** (CI: 0.24 – 0.47) | 0.35*** (0.24 – 0.49) | 0.38***(CI: 0.26 - 0.56) |
| Marital status | 0.88 (CI: 0.69-1.11) | 0.88 (CI:0 .68 – 1.18) | 0.86 (CI: 0.67 - 1.11) |
| Age | 0.95*** (CI: 0. 93 - 0.96) | 0.95***(CI: 0.93 -0.96) | 0.95***(CI: 0.93 - 0.96) |
| Number of Children | 1.96***(CI: 1.31 - 2.92) | 1.91**(CI: 1.27 - 2.86) | 1.75**(CI: 1.15 - 2.66) |
| Muslim Religion | 0.92 (CI: 0. .68- 1.24) | 0.95 (CI: 0.70 - 1.29) | 0.97 (CI: 0.70 - 1.34) |
| Education | 1.10 (CI: .98 - 1.23) | 1.10 (CI: 0.97 - 1.23) | 1.06 (CI: 0.93 - 1.20) |
| Awareness | 3.24** (CI: 1.54 - 6.81) | 3.09** (CI: 1.4 - 6.61) | 2.83** (CI: 1.29 - 6.21) |
| Descriptive norms |  | 1.29** (CI: 1.08-1.54) | 1.30** (CI: 1.08 - 1.57) |
| Injunctive norms |  | 0.75** (CI:0.63 - 0.88) | 0.73*** (CI:0.61 - 0.87) |
| Network contraceptive use |  |  | 2.54*** (CI: 1.69 - 3.82) |
| Network infertility beliefs |  |  | 0.81* (CI: 0.67 - 0.98) |
| Log Likelihood | -573.2 | -561.2 | -521.2 |
| (Pseudo R-squared) | (0.08) | (0.09) | (0.13) |

*Notes*: Infertility belief = individual belief that using contraception causes infertility. Awareness = awareness of different modern contraceptive methods. Descriptive norms = perceptions that people around you are using contraception. Injunctive norms = expectations that women should *not* be using contraception. Network contraceptive use = the average of modern contraceptive use of the 1-5 people that the respondent nominated. Infertility belief. Network infertility beliefs = the average belief that contraception use causes infertility of the 1-5 people that the respondent nominated.**p* < .05, ***p* < .01, ****p* < .001

**Appendix 2 Sensitivity Analysis – including all men & women regardless of marital status**

| ***Table 2. Odds ratios from logistic regression models predicting contraception use in married Kenyan men and women (N = 898)*** | | | |
| --- | --- | --- | --- |
|  | Individual-level model | Interpersonal-level model | Network-level model |
| Infertility belief | 0.82** (CI: 0.72-0.92) | 0.82** (CI: 0.71- 0.92) | 0.88 (CI: 0.77-1.01) |
| Village | 0.35*** (CI: 0.25 - 0.48) | 0.36*** (CI: 0.25-0. 50) | 0.42***(CI: .29- .59) |
| Marital status | 0.98 (CI: 0.82- 1.19) | 0.98 (CI: 0.81-1.18) | 1.01 (CI: 0.83 - 1.22) |
| Age | 0.96*** (CI: 0.94 - 0.97 | 0.96***(CI: 0.94 - 0.97) | 0.96*** (CI: 0.94-0.98) |
| Number of Children | 2.10*** (CI: 1.41 - 3.11) | 2.05***(CI: 1.37- 3.06) | 1.86** (CI: 1.23-2.80) |
| Muslim Religion | 0.74 (CI: 0.55- 1.00) | 0.76 (CI: 0.56-1.03) | 0.80 (CI:0.58 - 1.10) |
| Education | 1.20 (CI: 1.07- 1.34) | 1.20** (CI: 1.07-1.34) | 1.17** (CI: 1.04 - 1.31) |
| Awareness | 3.16** (CI: 1.55- 6.43) | 2.92** (CI: 1.40-6.05) | 2.63* (CI: 1.24 - 5.58) |
| Descriptive norms |  | 1.30** (CI: 1.09-1.53) | 1.29** (CI: 1.07-1.53) |
| Injunctive norms |  | 0.75*** (CI:0.64-0.88) | 0.74*** (CI: 0.62 -0 .87) |
| Network contraceptive use |  |  | 2.44*** (CI: 1.63 - 3.64) |
| Network infertility beliefs |  |  | 0.75** (CI:0.62 -.90) |
| Log Likelihood | -598.7 | -586.2 | -544.8 |
| (Pseudo R-squared) | (0.08) | (0.09) | (0.12) |

*Notes*: Infertility belief = individual belief that using contraception causes infertility. Awareness = awareness of different modern contraceptive methods. Descriptive norms = perceptions that people around you are using contraception. Injunctive norms = expectations that women should *not* be using contraception. Network contraceptive use = the average of modern contraceptive use of the 1-5 people that the respondent nominated. Network infertility beliefs = the average belief that contraception use causes infertility of the 1-5 people that the respondent nominated.**p* < .05, ***p* < .01, ****p* < .001.
